# Vemurafenib-resistant BRAF-V600E-mutated melanoma is regressed by MEK-targeting drug trametinib, but not cobimetinib in a patient-derived orthotopic xenograft (PDOX) mouse model

**DOI:** 10.18632/oncotarget.12328

**Published:** 2016-09-28

**Authors:** Kei Kawaguchi, Takashi Murakami, Bartosz Chmielowski, Kentaro Igarashi, Tasuku Kiyuna, Michiaki Unno, Scott D. Nelson, Tara A. Russell, Sarah M. Dry, Yunfeng Li, Fritz C. Eilber, Robert M. Hoffman

**Affiliations:** ^1^ AntiCancer Inc., San Diego, CA, USA; ^2^ Department of Surgery, University of California, San Diego, CA, USA; ^3^ Department of Surgery, Graduate School of Medicine, Tohoku University, Sendai, Japan; ^4^ Division of Hematology-Oncology, University of California, Los Angeles, CA, USA; ^5^ Department of Pathology, University of California, Los Angeles, CA, USA; ^6^ Division of Surgical Oncology, University of California, Los Angeles, CA, USA

**Keywords:** melanoma, PDOX, nude mice, orthotopic, drug-response

## Abstract

Melanoma is a recalcitrant disease. The present study used a patient-derived orthotopic xenograft (PDOX) model of melanoma to test sensitivity to three molecularly-targeted drugs and one standard chemotherapeutic. A BRAF-V600E-mutant melanoma obtained from the right chest wall of a patient was grown orthotopically in the right chest wall of nude mice to establish a PDOX model. Two weeks after implantation, 50 PDOX nude mice were divided into 5 groups: G1, control without treatment; G2, vemurafenib (VEM) (30 mg/kg); G3; temozolomide (TEM) (25 mg/kg); G4, trametinib (TRA) (0.3 mg/kg); and G5, cobimetinib (COB) (5 mg/kg). Each drug was administered orally, daily for 14 consecutive days. Tumor sizes were measured with calipers twice a week. On day 14 from initiation of treatment, TRA, an MEK inhibitor, was the only agent of the 4 tested that caused tumor regression (*P* < 0.001 at day 14). In contrast, another MEK inhibitor, COB, could slow but not arrest growth or cause regression of the melanoma. First-line therapy TEM could slow but not arrest tumor growth or cause regression. The patient in this study had a BRAF-V600E-mutant melanoma and would be considered to be a strong candidate for VEM as first-line therapy, since VEM targets this mutation. However, VEM was not effective. The PDOX model thus helped identify the very-high efficacy of TRA against the melanoma PDOX and is a promising drug for this patient. These results demonstrate the powerful precision of the PDOX model for cancer therapy, not achievable by genomic analysis alone.

## INTRODUCTION

Melanoma is a recalcitrant cancer. When melanoma metastasizes to regional lymph nodes, the 5-year survival rate is 29% and when it metastasizes to organs, the survival rate is 7% [1]. Although recently-developed immuno-therapy has extended survival to some extent, the 5-year survival rates have not been significantly increased [2]. Immuno-therapy involving PD-1/PD-L1 blockade has had some success with melanoma but is limited by lack of sufficient tumor infiltration of activated lymphocytes to kill the cancer cells within the tumor in the majority of patients tested [3]. Dacarbazine and cisplatinum have been used to treat melanoma with limited efficacy [4, 5].

Despite progress in melanoma therapy, there is still no cure for stage III and IV disease due to drug resistance, tumor heterogeneity and an immunosuppressed tumor microenvironment [2]. In addition, the presence of melanin appears to interfere with chemotherapy and radiotherapy of this recalcitrant disease [4]. Therefore, more effective approaches to melanoma treatment are needed.

Clinically-relevant mouse models of melanoma would permit evaluation of tailor-made individualized therapy based on the patient-derived tumor. Our laboratory pioneered the patient-derived orthotopic xenograft (PDOX) nude mouse model with the technique of surgical orthotopic implantation (SOI) [6]. Our laboratory has developed PDOX model of various types of tumors including pancreatic [7–10], breast [11], ovarian [12], lung [13], cervical [14], colon [15–17], stomach [18] and sarcoma [19–23]. Recent studies from our laboratory have demonstrated that PDOX models can be used to develop fluorescence-guided surgery [8, 17, 24], novel therapeutics such as tumor-targeting bacteria [21, 22, 25] and study the tumor microenvironment [26, 27]. The PDOX model, developed by our laboratory over the past 28 years, has many advantages over subcutaneous-transplant models which are growing ectopically under the skin [6].

The results of the present study indicate that molecular profiling alone may not predict drug response and that a PDOX model of the patient's tumor is necessary for precise individualized therapy.

## RESULTS AND DISCUSSION

**Figure 1:**
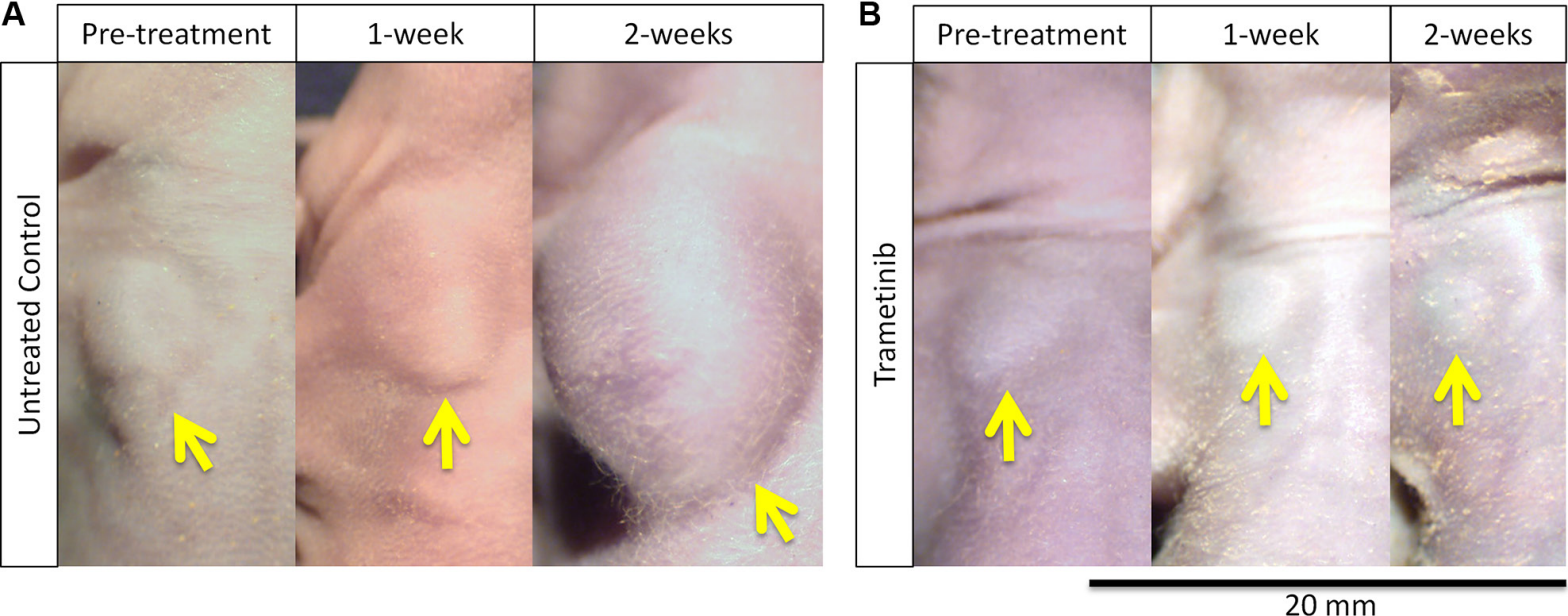
Melanoma regression caused by trametinib (TRA) in the PDOX model (**A**) The tumor size of the untreated control steadily increased. (**B**) The tumor treated with TRA steadily regressed. Yellow arrows show the PDOX tumor on the right chest wall. Scale bar: 20 mm.

All treatments significantly inhibited tumor growth compared to untreated control on day 14 after initiation: vemurafenib (VEM): *p* = 0.0117; temozolomide (TEM), *p* < 0.0001; trametinib (TRA), *p* < 0.0001; and cobimetinib (COB), *p* < 0.0001. However, tumor regression was observed only in the TRA group (Figure 1).

**Figure 2:**
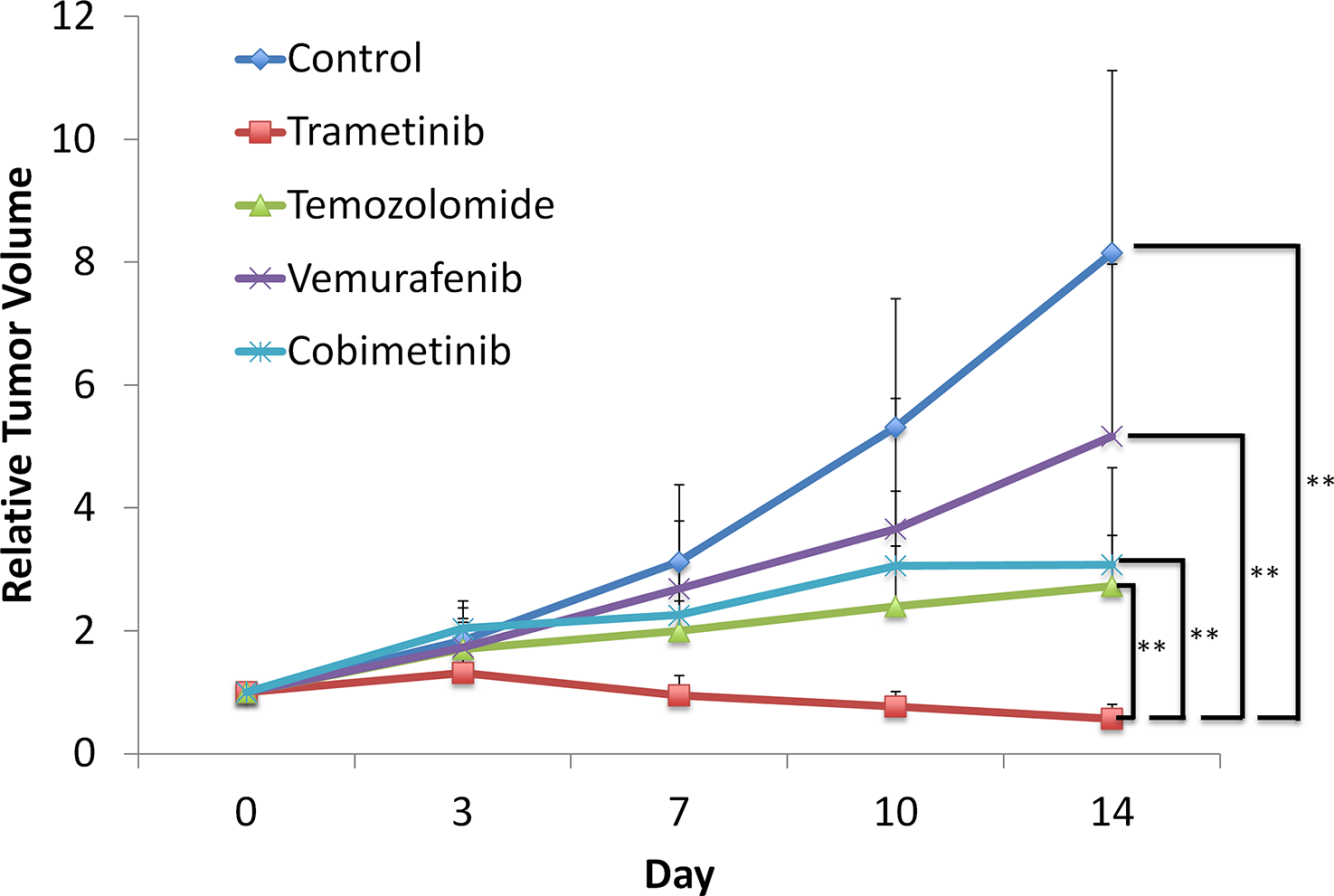
Trametinib (TRA) was the most effective agent against the melanoma PDOX model Line graph shows relative tumor volume at each point relative to the initial tumor volume. TRA significantly regressed the tumor compared to untreated control from day 3 to 14. From day 7, TRA was significantly more effective than all other therapies tested. *P* ≤ 0.0001. Error bars: ± SD.

As shown in Figure 2, TRA regressed tumor growth compared to untreated control from day 3 (day 3: *p* = 0.0171; day 7: *p* < 0.0001; day 10: *p* < 0.0001; day 14: *p* < 0.0001). On day 14, TRA showed significantly more efficacy compared to other therapies evaluated: VEM (*p* < 0.0001), COB (*p* = 0.0001), and TEM (*p* = 0.0001). The PDOX results suggest that TRA could be used as first-line therapy for this patient (Figure 2).

VEM, a tyrosine kinase inhibitor (TKI), targeting BRAF-V600E kinase, has been effective in the treatment of a BRAF-V600E-mutant melanoma [28–30]. The patient in the present study had a BRAF-V600E-mutant melanoma and would have been considered to be a strong candidate for VEM as first-line therapy. However, not all melanomas with mutant BRAF-V600E are responsive to VEM [28]. In the present study, VEM efficacy was significantly less than TRA (*p* < 0.0001), TEM (*p* = 0.0090) and COB (*p* = 0.0239), on day 14 (Figure 2).

**Figure 3:**
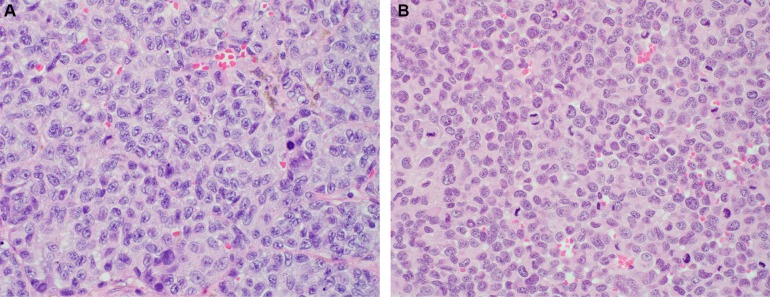
Histology of the original patient tumor and the untreated control PDOX tumor The histology of the PDOX untreated tumor closely matched the patient's tumor with the cells of both looking very similar. (**A**) Original patient tumor. (**B**) Untreated control PDOX tumor. See Materials and Methods for details.

The histology of the original patient tumor and the untreated PDOX tumor (Figure 3A, 3B) were similar, containing the same types of cells. However, nests of cancer cells are seen in the original, but not in the PDOX. Also, the original tumor was slightly melanotic, but the PDOX tumor did not appear to contain melanin. As mentioned above, high levels of melanin in melanoma may interfere with therapy [4].

COB is a MEK inhibitor, similar to TRA. It was reported that combination therapy of VEM and COB improved progression-free survival (PFS) in melanoma compared to VEM plus placebo [31]. COB mono-therapy was significantly more effective than VEM mono-therapy in our study (*p* = 0.0239). However, TRA was significantly more effective than COB (*p* < 0.0001) as discussed above.

TEM, an alkylating agent, had been widely used as a standard chemotherapy for melanoma. However, after approval of molecular-targeting agents, such as VEM, COB, and TRA, TEM is not usually the first choice for melanoma. However, not all melanomas have mutations that are targeted by these new agents and not all patients with these mutations are responsive to these drugs [28]. In our PDOX study, TEM was the most effective agent after TRA and significantly more effective than VEM (*p* = 0.0090), despite the patient having a BRAF-V600E mutation (Figure 2).

TRA is a MEK inhibitor, which has been shown to act downstream of KRAS, suppressing mitogen-activated protein kinase (MAPK) [32]. TRA was shown to affect the fibroblast growth factor receptor 1 (FGFR1) leading to drug resistance [32]. In a recent study, inhibition of FGFR1 in combination with TRA enhanced cancer cell death *in vitro* and *in vivo* [32]. The results of the present report, however, demonstrate that TRA was very active as a single agent in a BRAF-V600E mutant melanoma, that is only partially inhibited by VEM [30].

The results of the present study also demonstrate that drug response testing in the PDOX model can distinguish efficacy of drugs on individual tumors that have similar molecular targets. The results of the present study also indicate that molecular profiling alone may not predict drug response.

The histology of the PDOX untreated tumor closely matched the patient's tumor with the cells of both looking very similar (Figure 3), demonstrating the fidelity of the PDOX tumor.

## CONCLUSIONS

In the present study, the PDOX model helped identify the very high efficacy of TRA against the melanoma PDOX. TRA, a targeted MEK inhibitor, was the only agent of the 4 tested that caused tumor regression and is a promising drug for the patient donor of the PDOX. COB is also a targeted MEK inhibitor, but was only partially effective against the melanoma PDOX. VEM would have been predicted to be active against this tumor [30], which has a BRAF- V600E-mutation which VEM targets, but VEM was not effective. These results demonstrate the powerful precision of the PDOX model, not achievable by genomic analysis alone. TRA is a MEK inhibitor that also acts synergistically with agents that target the fibroblast growth factor receptor (FGFR1) such as ponatinib (PON) [32]. Future experiments will test the combination of TRA and PON in melanoma and other PDOX models. Future experiments will also compare the molecular action of TRA and COB to determine why they had a strong difference in efficacy in the present case.

Previously developed concepts and strategies of highly selective tumor targeting can take advantage of molecular targeting of tumors, including tissue-selective therapy which focuses on unique differences between normal and tumor tissues [33–38].

We recently reported that a PDOX model from another melanoma patient responded very well to tomur-targeting *Salmonella typhimurium* A1-R [39]. Future experiments will test the present and other melanoma PDOX with *Salmonella typhimurium* A1-R.

## MATERIALS AND METHODS

### Mice

Athymic *nu/nu* nude mice (AntiCancer Inc., San Diego, CA), 4–6 weeks old, were used in this study. All mouse surgical procedures and imaging were performed with the animals anesthetized by subcutaneous injection of a ketamine mixture (0.02 ml solution of 20 mg/kg ketamine, 15.2 mg/kg xylazine, and 0.48 mg/kg acepromazine maleate). The response of animals during surgery was monitored to ensure adequate depth of anesthesia. The animals were observed on a daily basis and humanely sacrificed by CO_2_ inhalation if they met the following humane endpoint criteria: severe tumor burden (more than 20 mm in diameter), prostration, significant body weight loss, difficulty breathing, rotational motion and body temperature drop. Animals were housed in a barrier facility on a high-efficacy particulate arrestance (HEPA)-filtered rack under standard conditions of 12-hour light/dark cycles. The animals were fed an autoclaved laboratory rodent diet. All animal studies were conducted in accordance with the principles and procedures outlined in the National Institutes of Health Guide for the Care and Use of Animals under Assurance Number A3873-1.

### Patient-derived tumor

A 75-year-old female patient was diagnosed with a melanoma of the right chest wall. The tumor was resected in the Department of Surgery, University of California, Los Angeles (UCLA). Written informed consent was provided by the patient, and the Institutional Review Board (IRB) of UCLA approved this experiment.

### Establishment of PDOX models of melanoma by surgical orthotopic implantation (SOI)

A fresh sample of the melanoma of the patient was obtained and transported immediately to the laboratory at AntiCancer, Inc., on wet ice. The sample was cut into 5-mm fragments and implanted subcutaneously in nude mice. After three weeks, the subcutaneously-implanted tumors grew to more than 10 mm in diameter. The subcutaneously-grown tumors were then harvested and cut into small fragments (3 mm^3^). After nude mice were anesthetized with the ketamine solution described above, a 5-mm skin incision was made on the right chest into the chest wall, which was split to make space for the melanoma tissue fragment. A single tumor fragment was implanted orthotopically into the space to establish the PDOX model. The wound was closed with a 6–0 nylon suture (Ethilon, Ethicon, Inc., NJ, USA) [23, 24].

### Treatment study design in the PDOX model of melanoma

PDOX mouse models were randomized into five groups of 10 mice each: G1, untreated control (*n* = 10); G2, treated with VEM (30 mg/kg, *n* = 10); G3, TEM (25 mg/kg, *n* = 10); G4, TRA (0.3 mg/kg, *n* = 10); G5, COB (5 mg/kg, *n* = 10) [1]. Each drug was administrated orally daily for 14 consecutive days. Tumor length and width were measured twice a week. Tumor volume was calculated with the following formula: Tumor volume (mm^3^) = length (mm) × width (mm) × width (mm) × 1/2. Data are presented as mean ± SD. The tumor volume ratio is defined at the tumor volume at any given time point relative to the initial tumor volume.

### Tumor histology

Tumor tissue was removed along with surrounding normal tissues at the time of resection from the patient and untreated control PDOX. The tissues were fixed in 10% formalin and embedded in paraffin before sectioning and staining. Tissue sections (3 μm) were deparaffinized in xylene and rehydrated in an ethanol series. Hematoxylin and eosin (H&E) staining was performed according to standard protocols. Histological examination was performed with a BHS system microscope. Images were acquired with INFINITY ANALYZE software (Lumenera Corporation, Ottawa, Canada).

### Statistical analysis

JMP version 11.0 was used for all statistical analyses. Significant differences for continuous variables were determined using the Mann-Whitney *U* test. Line graphs expressed average values and error bar showed SD. A probability value of *P* ≤ 0.05 was considered statistically significant.

## References

[1] Shain AH, Yeh I, Kovalyshyn I, Sriharan A, Talevich E, Gagon A, Dummer R, North J, Pincus L, Ruban B, Rickaby W, D'Arrigo C, Robson A (2015). The genetic evolution of melanoma from precursor lesions. N Engl J Med.

[2] Slominski AT, Carlson JA (2014). Melanoma resistance: a bright future for academicians and a challenge for patient advocates. Mayo Clin Proc.

[3] Tang H, Wang Y, Chlewicki LK, Zhang Y, Guo J, Liang W, Wang J, Wang X, Fu YX (2016). Facilitating T Cell infiltration in tumor microenvironment overcomes resistance to PD-L1 blockade. Cancer Cell.

[4] Brożyna AA, Jóźwicki W, Roszkowski K, Filipiak J, Slominski AT (2016). Melanin content in melanoma metastases affects the outcome of radiotherapy. Oncotarget.

[5] Flaherty LE, Othus M, Atkins MB, Tuthill RJ, Thompson JA, Vetto JT, Haluska FG, Pappo AS, Sosman JA, Redman BG, Moon J, Ribas A, Kirkwood JM (2014). Southwest Oncology Group S0008: a phase III trial of high-dose interferon Alfa-2b versus cisplatin, vinblastine, and dacarbazine, plus interleukin-2 and interferon in patients with high-riskmelanoma—an intergroup study of cancer and leukemia Group B, Children's Oncology Group, Eastern Cooperative Oncology Group, and Southwest Oncology Group. J Clin Oncol.

[6] Hoffman RM (2015). Patient-derived orthotopic xenografts: better mimic of metastasis than subcutaneous xenografts. Nature Reviews Cancer.

[7] Fu X, Guadagni F, Hoffman RM (1992). A metastatic nude-mouse model of human pancreatic cancer constructed orthotopically with histologically intact patient specimens. Proc Natl Acad Sci USA.

[8] Hiroshima Y, Maawy A, Zhang Y, Murakami T, Momiyama M, Mori R, Matsuyama R, Katz MH, Fleming JB, Chishima T, Tanaka K, Ichikawa Y, Endo I (2014). Metastatic recurrence in a pancreatic cancer patient derived orthotopic xenograft (PDOX) nude mouse model is inhibited by neoadjuvant chemotherapy in combination with fluorescence-guided surgery with an anti-CA 19–9-conjugated fluorophore. PLOS ONE.

[9] Hiroshima Y, Zhang Y, Murakami T, Maawy AA, Miwa S, Yamamoto M, Yano S, Sato S, Momiyama M, Mori R, Matsuyama R, Chishima T, Tanaka K (2014). Efficacy of tumor-targeting *Salmonella typhimurium* A1-R in combination with anti-angiogenesis therapy on a pancreatic cancer patient-derived orthotopic xenograph (PDOX) and cell line mouse models. Oncotarget.

[10] Hiroshima Y, Maawy AA, Katz MH, Fleming JB, Bouvet M, Endo I, Hoffman RM (2015). Selective efficacy of zoledronic acid on metastasis in a patient-derived orthotopic xenograph (PDOX) nude-mouse model of human pancreatic cancer. J Surg Oncol.

[11] Fu X, Le P, Hoffman RM (1993). A metastatic-orthotopic transplant nude-mouse model of human patient breast cancer. Anticancer Res.

[12] Fu X, Hoffman RM (1993). Human ovarian carcinoma metastatic models constructed in nude mice by orthotopic transplantation of histologically-intact patient specimens. Anticancer Res.

[13] Wang X, Fu X, Hoffman RM (1992). A new patient-like metastatic model of human lung cancer constructed orthotopically with intact tissue via thoracotomy in immunodeficient mice. Int J Cancer.

[14] Hiroshima Y, Zhang Y, Zhang M, Maawy A, Mii S, Yamamoto M, Uehara F, Miwa S, Yano S, Murakami T, Momiyama M, Chishima T, Tanaka K (2015). Establishment of a patient-derived orthotopic xenograph (PDOX) model of HER-2-positive cervical cancer expressing the clinical metastatic pattern. PLOS ONE.

[15] Fu X, Besterman JM, Monosov A, Hoffman RM (1991). Models of human metastatic colon cancer in nude mice orthotopically constructed by using histologically intact patient specimens. Proc Natl Acad Sci USA.

[16] Metildi CA, Kaushal S, Luiken GA, Talamini MA, Hoffman RM, Bouvet M (2014). Fluorescently-labeled chimeric anti-CEA antibody improves detection and resection of human colon cancer in a patient-derived orthotopic xenograft (PDOX) nude mouse model. J Surg Oncol.

[17] Hiroshima Y, Maawy A, Metildi CA, Zhang Y, Uehara F, Miwa S, Yano S, Sato S, Murakami T, Momiyama M, Chishima T, Tanaka K, Bouvet M (2014). Successful fluorescence-guided surgery on human colon cancer patient-derived orthotopic xenograft mouse models using a fluorophore-conjugated anti-CEA antibody and a portable imaging system. J Laparoendosc Adv Surg Tech A.

[18] Furukawa T, Kubota T, Watanabe M, Kitajima M, Fu X, Hoffman RM (1993). Orthotopic transplantation of histologically intact clinical specimens of stomach cancer to nude mice: correlation of metastatic sites in mouse and individual patient donors. Int J Cancer.

[19] Hiroshima Y, Zhang Y, Zhang N, Uehara F, Maawy A, Murakami T, Mii S, Yamamoto M, Miwa S, Yano S, Momiyama M, Mori R, Matsuyama R (2015). Patient-derived orthotopic xenograft (PDOX) nude mouse model of soft-tissue sarcoma more closely mimics the patient behavior in contrast to the subcutaneous ectopic model. Anticancer Res.

[20] Hiroshima Y, Zhao M, Zhang Y, Zhang N, Maawy A, Murakami T, Mii S, Uehara F, Yamamoto M, Miwa S, Yano S, Momiyama M, Mori R (2015). Tumor-targeting *Salmonella typhimurium* A1-R arrests a chemo-resistant patient soft-tissue sarcoma in nude mice. PLOS ONE.

[21] Murakami T, DeLong J, Eilber FC, Zhao M, Zhang Y, Zhang N, Singh A, Russell T, Deng S, Reynoso J, Quan C, Hiroshima Y, Matsuyama R (2016). Tumor-targeting *Salmonella typhimurium* A1-R in combination with doxorubicin eradicate soft tissue sarcoma in a patient-derived orthotopic xenograft PDOX model. Oncotarget.

[22] Kiyuna T, Murakami T, Tome Y, Kawaguchi K, Igarashi K, Zhang Y Zhao M, Li Y, Bouvet M, Kanaya F, Singh A, Dry S, Eilber FC, Hoffman RM (2016). High efficacy of tumor-targeting *Salmonella typhimurium* A1-R on a doxorubicin- and dactolisib-resistant follicular dendritic-cell sarcoma in a patient-derived orthotopic xenograft nude mouse model. Oncotarget.

[23] Murakami T, Singh AS, Kiyuna T, Dry SM, Li Y, James AW, Igarashi K, Kawaguchi K, DeLong JC, Zhang Y, Hiroshima Y, Russell T (2016). Effective molecular targeting of CDK4/6 and IGF-1R in a rare FUS-ERG fusion CDKN2A-deletion doxorubicin-resistant Ewing's sarcoma patient-derived orthotopic xenograft (PDOX) nude-mouse model. Oncotarget.

[24] Hiroshima Y, Maawy A, Sato S, Murakami T, Uehara F, Miwa S, Yano S, Momiyama M, Chishima T, Tanaka K, Bouvet M, Endo I (2014). Hand-held high-resolution fluorescence imaging system for fluorescence-guided surgery of patient and cell-line pancreatic tumors growing orthotopically in nude mice. J Surg Res.

[25] Hiroshima Y, Zhao M, Maawy A, Zhang Y, Katz MH, Fleming JB, Uehara F, Miwa S, Yano S, Momiyama M, Suetsugu A, Chishima T, Tanaka K (2014). Efficacy of *Salmonella typhimurium* A1-R versus chemotherapy on a pancreatic cancer patient-derived orthotopic xenograft (PDOX). J Cell Biochem.

[26] Suetsugu A, Katz M, Fleming J, Truty M, Thomas R, Saji S, Moriwaki H, Bouvet M, Hoffman RM (2012). Non-invasive fluorescent-protein imaging of orthotopic pancreatic-cancer-patient tumorgraft progression in nude mice. Anticancer Res.

[27] Suetsugu A, Katz M, Fleming J, Truty M, Thomas R, Saji S, Moriwaki H, Bouvet M, Hoffman RM (2012). Imageable fluorescent metastasis resulting in transgenic GFP mice orthotopically implanted with human-patient primary pancreatic cancer specimens. Anticancer Res.

[28] Chapman PB, Hauschild A, Robert C, Haanen JB, Ascierto P, Larkin J, Dummer R, Garbe C, Testori A, Maio M, Hogg D, Lorigan P, Lebbe C (2011). BRIM-3 Study Group. Improved survival with vemurafenib in melanoma with BRAF V600E mutation. N Engl J Med.

[29] Sosman JA, Kim KB, Schuchter L, Gonzalez R, Pavlick AC, Weber JS, McArthur GA, Hutson TE, Moschos SJ, Flaherty KT, Hersey P, Kefford R, Lawrence D (2012). Survival in BRAF V600-mutant advanced melanoma treated with vemurafenib. N Engl J Med.

[30] McArthur GA, Chapman PB, Robert C, Larkin J, Haanen JB, Dummer R, Ribas A, Hogg D, Hamid O, Ascierto PA, Garbe C, Testori A, Maio M (2014). Safety and efficacy of vemurafenib in BRAF(V600E) and BRAF(V600K) mutation-positive melanoma (BRIM-3): extended follow-up of a phase 3, randomised, open-label study. Lancet Oncol.

[31] Larkin J, Ascierto PA, Dréno B, Atkinson V, Liszkay G, Maio M, Mandalà M, Demidov L, Stroyakovskiy D, Thomas L, de la Cruz-Merino L, Dutriaux C, Garbe C (2014). Combined vemurafenib and cobimetinib in BRAF-mutated melanoma. N Engl J Med.

[32] Manchado E, Weissmueller S, Morris JP, Chen CC, Wullenkord R, Lujambio A, de Stanchina E, Poirier JT, Gainor JF, Corcoran RB, Engelman JA, Rudin CM, Rosen N (2016). A combinatorial strategy for treating KRAS-mutant lung cancer. Nature.

[33] Blagosklonny MV (2003). Matching targets for selective cancer therapy. Drug Discov Today.

[34] Blagosklonny MV (2005). Teratogens as anti-cancer drugs. Cell Cycle.

[35] Blagosklonny MV (2001). Treatment with inhibitors of caspases, that are substrates of drug transporters, selectively permits chemotherapy-induced apoptosis in multidrug-resistant cells but protects normal cells. Leukemia.

[36] Blagosklonny MV (2006). Target for cancer therapy: proliferating cells or stem cells. Leukemia.

[37] Apontes P, Leontieva OV, Demidenko ZN, Li F, Blagosklonny MV (2011). Exploring long-term protection of normal human fibroblasts and epithelial cells from chemotherapy in cell culture. Oncotarget.

[38] Blagosklonny MV (2003). Tissue-selective therapy of cancer. Br J Cancer.

[39] Yamamoto M, Zhao M, Hiroshima Y, Zhang Y, Shurell E, Eilber FC, Bouvet M, Noda M, Hoffman RM (2016). Efficacy of tomur-targeting *Salmonella typhimurium* A1-R on a melnoma patient-derived orthotopic xenograft (PDOX) nude-mouse model. PLoS One.

